# The silent spread: exploring diverse metastatic pathways in high-grade serous ovarian cancer

**DOI:** 10.3389/fmed.2025.1539024

**Published:** 2025-03-05

**Authors:** Mengqi Deng, Ruiye Yang, Junyi Jiang, Jinxu Zhang, Junqi He, Jinwei Miao

**Affiliations:** ^1^Beijing Obstetrics and Gynecology Hospital, Beijing Maternal and Child Health Care Hospital, Capital Medical University, Beijing, China; ^2^Laboratory for Clinical Medicine, Capital Medical University, Beijing, China; ^3^State Key Laboratory of Medical Proteomics, National Center for Protein Sciences (Beijing), Institute of Lifeomics, Beijing, China; ^4^Beijing Key Laboratory for Tumor Invasion and Metastasis, Department of Biochemistry and Molecular Biology, Capital Medical University, Beijing, China

**Keywords:** HGSOC, metastasis, immune microenvironment, lymphatic metastasis, hematogenous metastasis

## Abstract

High-grade serous ovarian cancer (HGSOC) is a highly aggressive and deadly gynecological cancer, with metastasis being a key factor in its poor prognosis. Historically, HGSOC was thought to spread primarily through the peritoneal cavity, but recent research has revealed additional routes of metastasis, including the blood and lymphatic systems. This review discusses the complex processes of HGSOC metastasis, focusing on peritoneal immune suppression, stromal reprogramming, and the role of circulating tumor cells in blood-based spread. We also explore the clinical significance of lymphatic metastasis, particularly its impact on patient outcomes. Gaining insight into molecular and genetic drivers, such as BRCA mutations and interactions within the immune microenvironment, is essential for developing targeted treatments. Future studies should aim to enhance experimental models, identify early detection markers, and investigate novel therapeutic approaches to effectively address HGSOC metastasis and improve patient survival.

## 1 Introduction

Ovarian cancer (OC) is the deadliest gynecological cancer, with most patients diagnosed at a late stage and quickly becoming resistant to chemotherapy, resulting in grim survival rates. Epithelial ovarian cancer (EOC) represents approximately 90% of all ovarian cancer cases, and it includes various subtypes, such as serous, endometrioid, clear cell, and mucinous subtypes, and additional categories. Among these, high-grade serous ovarian cancer (HGSOC) is the predominant histological variant and is often diagnosed at a late stage. Among advanced HGSOC patients, 80–100% exhibit peritoneal dissemination at the time of diagnosis, with frequent distant metastasis to other organs. Notably, metastasis to specific anatomical sites, such as the liver, lungs, and brain, is often associated with poorer clinical outcomes. Traditionally, OC, especially HGSOC, is thought to spread through the passive release of cancer cells into the abdominal cavity, facilitating surface-to-surface spread. However, clinical observations reveal that HGSOC follows a more complex metastatic pattern, with approximately two-thirds of advanced-stage HGSOC patients experiencing metastasis outside the peritoneum, suggesting the involvement of additional metastatic pathways (1). Thus, a comprehensive understanding of HGSOC metastasis mechanisms is crucial for improving treatment strategies.

Considering the metastatic pathways of HGSOC, it is essential to first address its origin, which remains a topic of ongoing debate. Initially, HGSOC was considered to start in the ovaries, but recent research over the last 20 years has identified serous tubal intraepithelial carcinoma (STIC) in the fallopian tubes as a potential precursor. However, pure STIC lesions are rarely observed in sporadic HGSOC, and many HGSOC cases do not show STIC lesions, leading to alternative hypotheses regarding the tumor’s origin, including other cell types, stromal fibroblasts, or even polyploid giant cancer cells (2).

In approximately 50% of HGSOC cases, mutations in genes related to homologous recombination (HR) repair, including BRCA1 and BRCA2, result in HR deficiency (3). HGSOC with BRCA mutations may have a greater tendency to metastasize to pelvic organs and the brain, indicating that genetic factors could play a role in determining tumor spread (4). Furthermore, HR deficiency not only hinders DNA repair but may also alter the tumor’s immune microenvironment, impacting immune surveillance and the ability of cancer to metastasize (4). The variation in mutation profiles between primary and secondary tumors suggests that the metastatic process and tumor progression are influenced by the specific microenvironments at different sites, suggesting that metastasis is a multifaceted process rather than a straightforward one (5). This review critically evaluates the existing evidence on the metastatic routes of HGSOC, including the peritoneal, hematogenous, and lymphatic pathways.

## 2 Peritoneal metastasis

**Figure 1:**
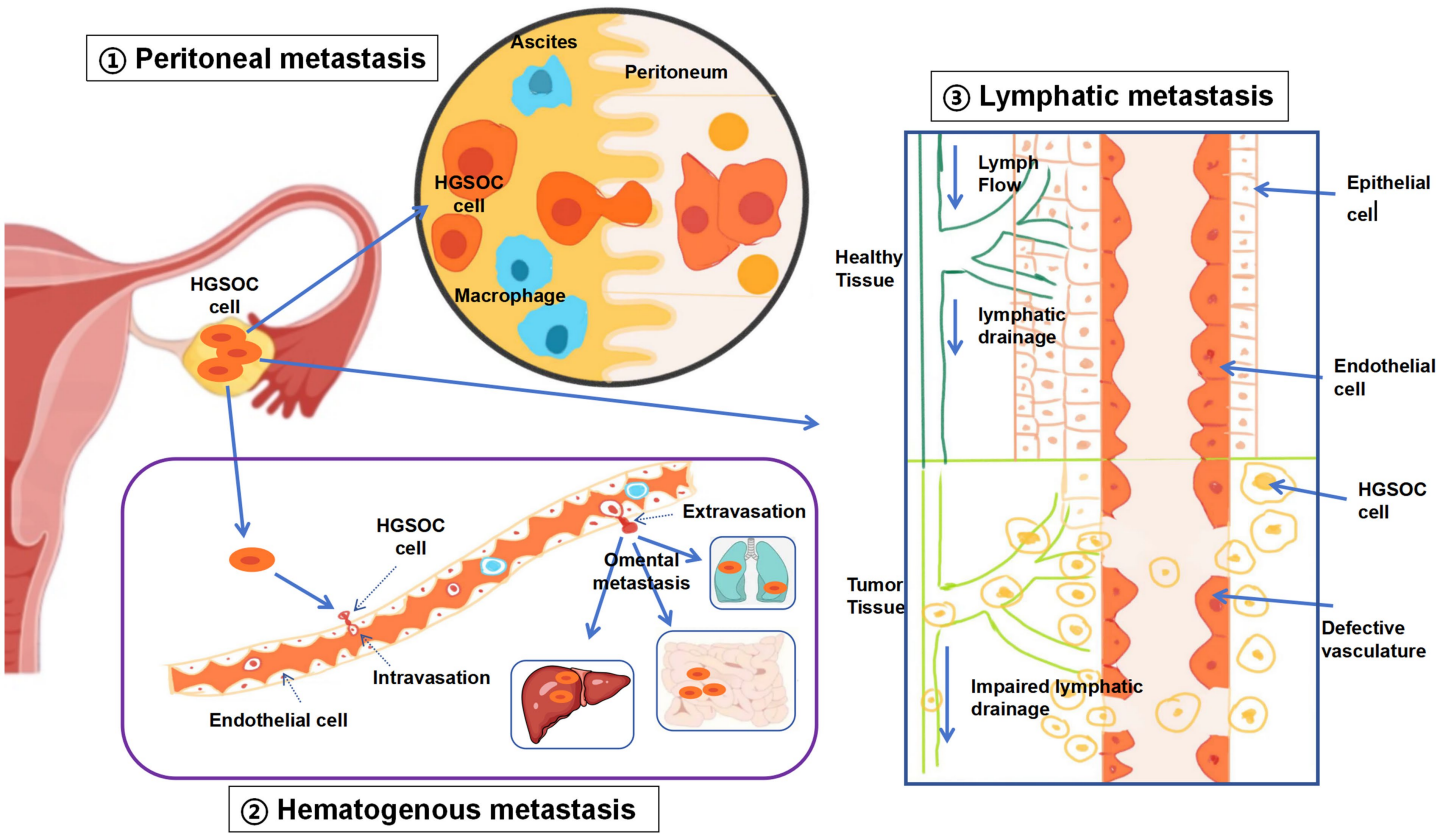
Three modes of metastasis of HGSOC. Peritoneal metastasis: traditionally, peritoneal metastasis was regarded as the primary pathway for EOC to spread. This occurs when ovarian cancer cells are discharged into the abdominal cavity, often facilitated by the presence of ascites. Hematologic metastasis: in hematogenous metastasis, EOC cells circulate through the bloodstream, reaching remote sites such as the liver, mesentery, or omentum, without involving the peritoneum. Lymphatic metastasis: the dissemination of EOC via the lymphatic system entails cancer cells infiltrating lymphatic vessels, moving to lymph nodes, and potentially spreading to other organs once they enter the blood circulation. Following lymph node involvement, EOC cells can further propagate through the lymphatic network to additional organs.

For many years, it has been widely believed that ovarian cancer spreads primarily by detaching from the original tumor and dispersing into the abdominal cavity, with the predominant mode of dissemination being transfer from one surface to another (Figure 1). Specifically, malignant cells detach from the initial tumor, travel through the abdominal space, and then establish themselves in different organs and tissues within that area. The fluid flow within the peritoneal cavity, typically in a clockwise direction, may influence the sites of cancer cell colonization, which explains the higher incidence of implantation in the upper right quadrant of the abdomen than in the upper left quadrant. For peritoneal dissemination, HGSOC cells need to bypass anoikis to embed effectively and penetrate the mesothelial lining. However, alternative mechanisms for peritoneal spread must also be considered (6).

HGSOC tumors exhibit two distinct morphological patterns in patients: type I, characterized by deep infiltration, millet-like lesions, and surrounding tissue distortion, and type II, characterized by exophytic nodules with a superficial appearance. Each type may be associated with different metastatic pathways. A critical element impacting the development and expansion of cancer cell groups is the existence of a premetastatic environment. This specialized microenvironment is typically composed of adipocytes, such as the omentum, which is widely recognized as the preferential site for HGSOC metastasis via either direct diffusion or hematogenous routes (7). The interactions between adipocytes and cancer cells, along with the cytokines secreted by adipocytes, promote the metastasis and growth of ovarian cancer cells (7, 8). Additionally, various immune-related determinants support organ tropism at the premetastatic niche, such as macrophages, extracellular vesicles, and particles, all of which can influence cancer cell metastasis and the establishment of the metastatic niche through multiple mechanisms (8).

The widespread dissemination of ovarian cancer to the parietal and visceral peritoneum is one of the most common forms of metastasis. Studies have shown that peritoneal mesothelial cells (PMCs), which act as a mechanical barrier, can prevent tumor cells from adhering to and invading underlying tissues (9, 10). However, exosomes secreted by ovarian cancer cells can directly or indirectly influence the peritoneum through ascitic fluid, mediating the formation of local PMN immune suppression. These immune suppression factors alter the vascular state of the tissue, ultimately enhancing the adhesion, implantation, invasiveness, and widespread peritoneal dissemination of ovarian cancer cells (9, 10).

### 2.1 Peritoneal local immune cell recruitment and dysfunction

Peritoneal immune suppression (PMN) in ovarian cancer is characterized by the dysfunction of dendritic cells (DCs), a reduction in CD8+ T-cell numbers, an increase in myeloid-derived suppressor cells (MDSCs), and alterations in immune cell phenotypes (11, 12). Chen et al. (11) reported that DCs in the PMN of ovarian cancer patients exhibit excessive activation of the endoplasmic reticulum stress factor XBP1, disrupting intracellular lipid homeostasis, which impairs their antigen-presenting function and hinders the activation and proliferation of tumor-killing T cells. Taki et al. (12) demonstrated that ovarian cancer-derived CXCL1 and CXCL2 recruit immune-suppressive MDSCs from the bone marrow into the peritoneum, significantly inhibiting CD8+ T-cell proliferation. Additionally, ovarian cancer-secreted exosomes regulate the p53-*β*-catenin-CCL2 signaling pathway to induce CCL2 production, which recruits macrophages to the peritoneum and promotes their polarization from the M1 phenotype to the M2 phenotype, thereby facilitating PMN-mediated immune suppression (10).

### 2.2 Abnormal neovascularization in the peritoneum

PMN-associated immune suppression also facilitates cancer cell extravasation, adhesion, survival, proliferation, and immune evasion through alterations in the vascular environment (13). The formation of new blood vessels within the PMN involves the disruption of the vascular system, endothelial cell migration, and lumen formation. Kakogiannos et al. (14) reported that, compared to healthy controls, ovarian cancer patients exhibit the downregulation of junctional adhesion molecule-A (JAM-A) and the tight junction protein claudin-5 in their blood vessels, which weakens endothelial tight junctions and increases vascular permeability. Furthermore, Mezouar et al. (15) reported that soluble E-cadherin (sE-cad), which is secreted by ovarian cancer cells, stimulates endothelial cell migration to the metastatic site to form new blood vessels while increasing vascular permeability, which promotes ovarian cancer cell metastasis and malignant ascites formation.

### 2.3 Acquisition of cancer cell invasiveness and stem cell properties

The PMN attracts free-floating cancer cells from ascitic fluid and circulating tumor cells (CTCs), facilitating their adhesion and colonization. Moreover, PMN-induced immune suppression leads to enhanced invasiveness and the acquisition of stem-like properties in ovarian cancer cells. Ovarian cancer-secreted TGF-β1 induces the expression of the cancer-associated fibroblast (CAF) marker fibronectin in peritoneal mesothelial cells (PMCs), which facilitates the adhesion and colonization of cancer cells. Exosomes secreted by ovarian cancer cells carry ETS1, which promotes the M2 polarization of peritoneal macrophages, contributing to PMN immune suppression (16, 17). Notably, TGF-beta has been shown to enhance cancer cell invasiveness by modulating the epithelial-to-mesenchymal transition (EMT) pathway (16). Furthermore, recent evidence demonstrates that TGF-beta signaling can promote the acquisition of stem-like properties in ovarian cancer cells by activating key pathways such as SMAD and non-SMAD signaling, which are implicated in stemness and drug resistance (17). These exosomes also secrete CXCL5 and CCL2, which interact with receptors on tumor cells to accelerate the peritoneal metastasis of ovarian cancer (18). Studies have shown that ovarian cancer cells adhering to the PMN undergo antiapoptotic transformation, shifting to a proliferative state, suppressing apoptosis, and promoting the invasion of disseminated cancer cells (19). For example, ovarian cancer exosomes carrying miRNA-21 inhibit apoptosis and increase proliferation at metastatic sites, thereby increasing invasiveness (20). Additionally, tumor cells shed from ovarian cancer ascites express several cancer stem cell markers on their surface, including CD44, CD54, CD55, CD133, and CD117, with significant upregulation of metastasis-related genes (21).

### 2.4 Inflammation and stromal reprogramming in the peritoneum

PMNs also exhibit inflammation and stromal reprogramming, both of which are crucial for cancer cell colonization, survival, and extensive peritoneal metastasis (22). Under stress, macrophages in the PMN release a variety of proinflammatory factors, such as TNF-*α*, MIP-2/CXCL2, MIP-1β/CCL4, CCL2/MCP-1, sICAM-1/CD54, and G-CSF, all of which promote tumor activity (23). Gartung et al. (24) demonstrated that dual inhibition of cyclooxygenase 2 (COX-2) and soluble epoxide hydrolase (sEH) suppresses the release of these proinflammatory factors, slows ovarian cancer progression, and prolongs patient survival. Additionally, CAFs undergo metabolic reprogramming in response to tumor stimuli, with significantly increased expression of arachidonic acid metabolic genes, which promote the production of inflammatory mediators and reshape the tumor PMN (24, 25). Compared to normal fibroblasts, CAFs exhibit increased expression of glycolytic metabolic genes, leading to excessive lactate production in the PMN. Tumor cells in proximity can directly take up lactate to promote growth, which further supports cancer cell colonization (25).

## 3 Hematogenous metastasis

Unlike the transperitoneal spread of ovarian cancer, the mechanisms underlying hematogenous and lymphatic metastasis are not yet fully understood, largely owing to the scarcity of suitable experimental models (26) and the lack of comprehensive clinical data regarding blood and lymphatic spread (Figure 1). As a result, the importance of metastasis through the bloodstream might be underappreciated. While hematogenous and lymphatic metastases seldom cause instant fatality, they are strongly linked to a grim prognosis (27). As therapeutic methods improve and survival rates for ovarian cancer patients increase, there is a corresponding rise in the likelihood of distant metastasis, particularly in patients with longer survival times. This is because prolonged survival provides more opportunity for cancer cells to disseminate and establish secondary tumors (28). Furthermore, ovarian cancer cells can spread through the bloodstream, showing a marked inclination toward the omentum (29), the most frequent location where ovarian cancer metastasizes. These findings indicate that blood-borne dissemination might be a crucial mechanism in the development of peritoneal metastasis.

The hematogenous spread has been recognized as clinically significant only in recent years. The evidence of retroperitoneal, submesothelial, and distant metastases cannot be solely attributed to transperitoneal spread (30). The detection of circulating tumor cells (CTCs) in the bloodstream of ovarian cancer patients strongly indicates the involvement of blood-borne routes in distant metastasis (31). Additionally, the role of the lymphatic system in the spread of ovarian cancer is recognized in the International Federation of Gynecology and Obstetrics (FIGO) staging criteria, with a clear link to unfavorable outcomes (32). In epithelial ovarian cancer (EOC), distant metastases, such as those to the liver, spleen, lungs, and gastrointestinal tract, often indicate a hematogenous mechanism. Even when the peritoneal lining over these metastatic sites is undamaged, blood-borne dissemination can occur without the need for peritoneal penetration.

Research utilizing syngeneic mouse models has demonstrated that EOC cells predominantly spread to the omentum via the bloodstream, a process enhanced by specific signaling pathways, such as the ErbB3–NRG1 axis. These studies highlight the ErbB3/NRG1 signaling pathway as a critical mediator of hematogenous metastasis to the omentum (33). When CXCR4 expression was reduced, there was a notable decrease in the number of circulating ovarian cancer cells, indicating that the SDF1/CXCR4 pathway might also be involved in blood-borne dissemination (33, 34). Furthermore, CTCs are frequently found in the blood of EOC patients, suggesting their involvement in hematogenous spread (34). Nevertheless, the survival rate of CTCs is low due to challenges such as shear stress and difficulties in extravasation, which may limit the effectiveness of hematogenous spread compared to direct diffusion (35). Importantly, despite the development of various techniques for capturing and analyzing CTCs, a combination of antibodies should be used to ensure the capture of all CTC types, including those that have undergone epithelial-to-mesenchymal transition (EMT), not just those expressing epithelial markers, such as vimentin, N-cadherin, and fibronectin.

## 4 Lymphatic metastasis

Lymphatic metastasis is commonly observed in patients with epithelial ovarian cancer (EOC). The ovarian tissue is rich in lymphatic vessels, which transport cancer cells to regional lymph nodes, such as the para-aortic and paracaval nodes, pelvic iliac nodes, and inguinal nodes (Figure 1). These lymph nodes serve as the initial sites of lymphatic drainage, although metastasis may also occur in more distant lymph nodes, such as those in the mediastinum and supraclavicular regions. EOC metastasis through the lymphatic system occurs when malignant cells infiltrate lymphatic channels, travel to lymph nodes, and possibly spread to remote organs via the circulatory system. The exact pathway by which metastatic EOC cells reach the bloodstream—directly from the primary tumor or after establishing themselves in the lymph nodes—remains uncertain. Furthermore, the inherent rhythmic contractions of lymphatic vessels contribute to lymphatic fluid movement, potentially facilitating the dissemination of cancer cells within the lymphatic network (36).

Several molecular factors have been implicated in ovarian cancer lymph node metastasis, including USP7, FAK, and the VEGFC-VEGFR3 signaling axis, all of which are associated with an increased incidence of lymphatic metastasis in ovarian cancer patients (26).

The lymph nodes draining the tumor contain diverse populations of immune cells whose interactions with cancer cells may influence tumor invasion and colonization. After reaching the sentinel lymph node—the first lymph node encountered in the tumor drainage pathway—EOC cells are capable of evading immune surveillance through various mechanisms (37). These processes encompass the variability in tumor antigens, the impairment of T-cell function by tumors, and the release of cytokines that suppress immune responses, facilitating the successful establishment of cancer cells in the lymph nodes. However, the precise mechanisms driving EOC lymphatic metastasis remain poorly understood.

Clinical evidence underscores the significant role of the lymphatic pathway in HGSOC dissemination, with lymph node-positive patients typically experiencing a worse prognosis. Interestingly, compared to peritoneal metastasis, spread to pelvic and para-aortic lymph nodes is linked with more positive clinical outcomes (28). The presence of tumor cells within the lymphatic or blood vessel channels of the primary tumor, known as lymphovascular space invasion (LVSI), is associated with poor clinical outcomes for ovarian cancer patients (26). As the initial phase of cancer cell dissemination into the bloodstream or lymphatic system, LVSI signifies a greater likelihood of both hematogenous and lymphatic metastasis.

However, clinical data regarding blood and lymphatic spread are frequently complicated by multiple elements, as malignant cells may spread through multiple pathways simultaneously. Additionally, relevant experimental models, such as syngeneic models (38), are complex and difficult to implement. Consequently, although the processes behind this new metastatic route in ovarian cancer hold substantial clinical importance, they continue to be enigmatic and difficult to pinpoint.

## 5 The application of emerging techniques in HGSOC metastasis

### 5.1 Liquid biopsy

Liquid biopsy, a minimally invasive approach to analyzing circulating tumor-derived components, has emerged as a transformative tool in studying HGSOC metastasis. By detecting circulating tumor DNA (ctDNA), circulating tumor cells (CTCs), and exosomes, this technology provides real-time insights into tumor dynamics and metastatic progression (39). For instance, David et al. demonstrated that ctDNA levels in plasma correlate with tumor burden and predict poor prognosis in HGSOC patients, particularly in cases of peritoneal metastasis (40). Additionally, CTCs expressing CD44 isolated from patients have been shown to exhibit chemoresistance and multipotency *ex vivo* (41). Recent advances in exosome analysis further highlight the role of tumor-derived exosomes in preparing the metastatic niche (42). A study by Chen et al. revealed that HGSOC exosomes carry immunosuppressive miRNAs (e.g., miR-21-3p, miR-125 b-5p, and miR-181 d-5p), which reprogram macrophages toward a pro-tumorigenic phenotype, facilitating immune evasion in distant organs (43). Future directions may focus on integrating multi-analyte liquid biopsy panels (e.g., combining ctDNA, CTCs, and exosomal proteins) to improve sensitivity and specificity in detecting occult metastasis.

### 5.2 3D organoid models

Three-dimensional (3D) organoid models have revolutionized the study of tumor metastasis by recapitulating the complex architecture and cellular interactions of the tumor microenvironment (44). These models, derived from patient tumor samples or cell lines, enable researchers to investigate HGSOC metastasis mechanisms in a physiologically relevant context (45). Recent studies have utilized 3D organoids to simulate peritoneal metastasis, a dominant route of HGSOC dissemination (46). These models also serve as a platform for high-throughput drug screening (47). Furthermore, using an organoid patient-derived model, they showed that low sTIMs were significantly associated with an increased response to anti-PD-1 treatment, indicating that mast cells could represent a novel immune target in HGSOC (48). Future advancements may integrate microfluidic systems to dynamically model the mechanical stresses encountered during hematogenous or lymphatic spread, further bridging the gap between *in vitro* models and clinical realities.

## 6 Future directions

Several key areas warrant further investigation. First, there is a need for more comprehensive studies on the molecular and genetic drivers of metastasis, particularly in the context of ovarian cancer mutations, such as BRCA1/2 and homologous recombination deficiencies, which may predispose tumors to specific metastatic patterns. Moreover, investigating the processes that enable tumors to evade the immune system and adapt to various microenvironments is essential, as this could reveal new therapeutic targets. Sophisticated models, such as 3D organoids and patient-derived xenografts, which can mimic the intricate nature of HGSOC metastasis, are vital for understanding the interplay between cancer cells and their metastatic surroundings. Additionally, the discovery of biomarkers linked to early-stage metastasis, especially those that can be identified in blood or ascitic fluid, could significantly increase early detection and treatment surveillance.

## 7 Conclusion

In summary, while significant advancements have been made in deciphering the metastatic mechanisms of HGSOC, further study is still necessary. Further investigations into the genetic, molecular, and immunological elements that fuel metastasis, along with the creation of novel treatment strategies aimed at these pathways, could enhance therapeutic efficacy and increase survival rates for individuals afflicted by this aggressive cancer.
